# A pilot study of a novel method to visualize three-dimensional dose distribution on skin surface images to evaluate radiation dermatitis

**DOI:** 10.1038/s41598-022-06713-7

**Published:** 2022-02-17

**Authors:** Ye-In Park, Seo Hee Choi, Chae-Seon Hong, Min-Seok Cho, Junyoung Son, Ji Won Jang, Jihun Kim, Hojin Kim, Dong Wook Kim, Jin Sung Kim

**Affiliations:** 1 Department of Radiation Oncology, Yonsei Cancer Center, Yonsei University College of Medicine, 50-1 Yonsei-ro, Seodaemun-gu, Seoul, South Korea; 2 Department of Radiation Oncology, Yongin Severance Hospital, Yonsei University College of Medicine, Yongin, South Korea

**Keywords:** Radiotherapy, Radiotherapy, Risk factors, Quality of life, Three-dimensional imaging

## Abstract

Predicting the radiation dose‒toxicity relationship is important for local tumor control and patients’ quality of life. We developed a first intuitive evaluation system that directly matches the three-dimensional (3D) dose distribution with the skin surface image of patients with radiation dermatitis (RD) to predict RD in patients undergoing radiotherapy. Using an RGB-D camera, 82 3D skin surface images (3DSSIs) were acquired from 19 patients who underwent radiotherapy. 3DSSI data acquired included 3D skin surface shape and optical imaging of the area where RD occurs. Surface registration between 3D skin dose (3DSD) and 3DSSI is performed using the iterative closest point algorithm, then reconstructed as a two-dimensional color image. The developed system successfully matched 3DSSI and 3DSD, and visualized the planned dose distribution onto the patient's RD image. The dose distribution pattern was consistent with the occurrence pattern of RD. This new approach facilitated the evaluation of the direct correlation between skin-dose distribution and RD and, therefore, provides a potential to predict the probability of RD and thereby decrease RD severity by enabling informed treatment decision making by physicians. However, the results need to be interpreted with caution due to the small sample size.

## Introduction

Radiation dermatitis (RD) is one of the most common acute toxicities associated with radiotherapy. Severe acute toxicities can negatively affect treatment outcomes, including the quality of life, subsequently leading to radiotherapy discontinuation^1,2^. Skin toxicity is a limiting factor in the radiation dose required to achieve local tumor control^3^. Therefore, accurate prediction of the radiation dose-toxicity relationship is important to ameliorate the skin toxicity profile while delivering the appropriate target dose.

The occurrence of RD is associated with several factors such as dosimetric parameters, chemotherapy, patient-related factors, and treatment techniques^4–8^. In particular, Dosimetric factors have been used to identify factors associated with RD occurrence as main indicators predicting skin toxicity^9,10^. An objective evaluation and documentation of the degree of RD are first needed. The level of RD may be assessed using the Common Terminology Criteria for Adverse Events and Radiation Therapy Oncology Group grading scales^11,12^, a subjective and qualitative analysis. Quantitative methods to document RD objectively use visual tools, such as cameras^13–15^. However, these remain insufficient for direct assessment of the correlation between RD and radiation dose. The heterogeneous dose distribution and partial hot spots may lead to acute skin toxicity. Intensity-modulated radiotherapy (IMRT) and volumetric-modulated arc therapy (VMAT) effectively reduce the risk of developing severe RD but may render the skin dose distribution non-uniform^9,16^. RD may vary in severity even within the individual patient's irradiated field. Thus, quantitatively analyzing the relationship between RD and the heterogeneous skin dose-volume distribution is an essential step in predicting RD occurrence.

New treatment techniques in radiotherapy include particle therapy and hypofractionated radiotherapy. As treatment techniques and the treatment planning system (TPS) dose calculation accuracy differ across institutions, there are limits to predicting RD by using common predictors^17^. This means that dosimetric predictors that correlate with severe RD in one institution may not be associated with RD patterns in another institution. Therefore, to use predictors reported by individual institutions in clinical practice, it is necessary to verify the accuracy and acceptability of the predictors. To improve RD outcomes, a treatment plan to minimize an RD-related dose is necessary. An accurate correlation between the TPS-calculated dose distribution and RD may improve RD management through the TPS-calculated dose. If the dose distribution corresponding to the area of RD occurrence can be quantitatively identified, treatment plans in patients with a high risk of RD may be adjusted accordingly and patients may be better informed about the expected toxicity.

To achieve a predictive model, it is necessary to match the skin dose distribution calculated by the TPS and RD occurrence images. Dosimetric factors and skin dose-volume histograms are proposed as prognostic measures for radiation-induced toxicity^10,18–20^. However, they are not suitable for determining the effect of the TPS dose-distribution pattern on skin toxicity within the irradiation field. To the best of our knowledge, there have been no studies that directly matched the three-dimensional skin dose (3DSD) distribution with RD images.

We present a method to visualize the radiation dose distribution on RD images. Here, we developed a new radiation dose-toxicity evaluation system (RaTES) that directly matches the 3D dose distribution calculated by the TPS with the 3D skin surface image (3DSSI) of RD in patients to obtain a reasonable prediction of RD in patients undergoing radiotherapy. This study provides an important method for accurately predicting and managing RD at the treatment planning stage by evaluating the direct correlation between skin dose distribution and RD severity with respect to morphology.

## Results

We collected 82 3DSSIs from 19 patients who underwent radiotherapy. These images were used to validate the RaTES. We included 8 head and neck, 6 breast, and 5 thoracic cancer patients. All patients (excluding Patient 13) were treated with VMAT. Patient 13 was treated with 3D conformal radiotherapy, and Patients 8 and 13 were treated with a combination of photon and electron beams. Five patients (Patients 1, 2, 8, 12, and 13) were treated with a tissue-equivalent bolus material to ensure acceptable target coverage. All patients were treated using 6 MV in the case of photon beam therapy; patients treated with a photon and electron beam combination were treated with 6 MeV (Patient 8) and 9 MeV (Patient 13). Patient and treatment characteristics are summarized in Tables 1 and 2.

**Table 1:** Patient characteristics.

Patient number	Age (years)	Sex	Tumor site	Histology	TNM stage	Chemotherapy	Hormonal therapy	Smoker
Pt 1	67	Female	Breast	IDC	cT2N2M1	Yes	No	No
Pt 2	73	Female	Breast	ILC&IDC	pT2N3M0	Yes	Yes	No
Pt 3	75	Female	Oral cavity	SCCa	pT1N0M0	No	No	No
Pt 4	60	Male	Nasopharynx	PD	cT1N2M0	Yes	No	No
Pt 5	42	Male	Lung	Small cell	cT1cN2M0	Yes	No	No
Pt 6	64	Male	Esophagus	SCCa	cTxN2M1	Yes	No	Ex-smoker
Pt 7	76	Female	Parotid gland	EMC	pT2N2M1	No	No	No
Pt 8	72	Female	Lung	AdenoCa	pT2aN0M0	No	No	No
Pt 9	63	Male	Esophagus	SCCa	cT3N2/3M0	Yes	No	Yes
Pt 10	88	Male	Larynx	SCCa	pT2N0M0	No	No	Ex-smoker
Pt 11	44	Female	Breast	ILC	pT2N1M0	Yes	Yes	No
Pt 12	81	Female	Breast	IDC	pT2N1M0	Yes	No	No
Pt 13	68	Female	Breast	IDC	cT2N2M1	Yes	No	No
Pt 14	58	Male	Larynx	SCCa	cT3N0M0	Yes	No	Yes
Pt 15	72	Male	Lung	SCCa	cT4N0M0	Yes	No	Ex-smoker
Pt 16	58	Male	Oropharynx	SCCa	pT2N2M0	Yes	No	Yes
Pt 17	57	Male	Tongue	SCCa	pT3N2bM0	Yes	No	Yes
Pt 18	64	Male	Hypopharynx	SCCa	cT1N1M1	Yes	No	No
Pt 19	74	Female	Breast	IDC	cT4N3M1	Yes	No	No

*IDC* Invasive ductal carcinoma, *ILC* invasive lobular carcinoma, *SCCa* squamous cell carcinoma, *PD* poorly differentiated, *EMC* epithelial myoepithelial carcinoma, *AdenoCa* adenocarcinoma.

**Table 2:** Patient treatment specifications and skin toxicity.

Patient number	Prescription dose (cGy)	Total # fractions	Treatment technique	Energy	# Arcs (beams)	Bolus	3DSSI imaged	Toxicity^a^ (CTCAE)
Pt 1	4500	15	VMAT	6 MV	2	Yes	2	G3
Pt 2	4005	15	VMAT	6 MV	2	Yes	3	G1
Pt 3	5250	25	VMAT	6 MV	2	No	5	G2
Pt 4	6950	15	VMAT	6 MV	2	No	4	G2
Pt 5	6000	30	VMAT	6 MV	2	No	5	G0
Pt 6	6300	30	VMAT	6 MV	2	No	5	G3
Pt 7	6000	30	VMAT	6 MV	1	No	7	G2
Pt 8	5000	25	VMAT/Electron^b^	6 MV/6 MeV^c^	3	Yes	5	G1
Pt 9	6300	30	VMAT	6 MV	2	No	8	G3
Pt 10	6525	29	VMAT	6 MV	1	No	5	G1
Pt 11	4005	15	VMAT	6 MV	2	No	2	G1
Pt 12	4005	15	VMAT	6 MV	2	Yes	4	G1
Pt 13	3750	15	3DCRT/Electron	6 MV/9 MeV	3	Yes	4	G3
Pt 14	6750	30	VMAT	6 MV	2	No	5	G2
Pt 15	6000	30	VMAT	6 MV	2	No	3	G0
Pt 16	2880	14	VMAT	6 MV	2	No	3	G0
Pt 17	6300	30	VMAT	6 MV	2	No	4	G2
Pt 18	6996	33	VMAT	6 MV	2	No	6	G2
Pt 19	4500	15	VMAT	6 MV	2	No	2	G1

*VMAT* volumetric- modulated arc therapy, *3DCRT* three-dimensional conformal radiotherapy, *3DSSI* 3D skin surface image, *CTCAE* Common Terminology Criteria for Adverse Events.

^a^Radiation dermatitis was graded according to the CTCAE v4.0.

^b^Combination of VMAT and electron irradiation.

^c^6 MV photon and 6 MeV Electron beams.

**Figure 1:**
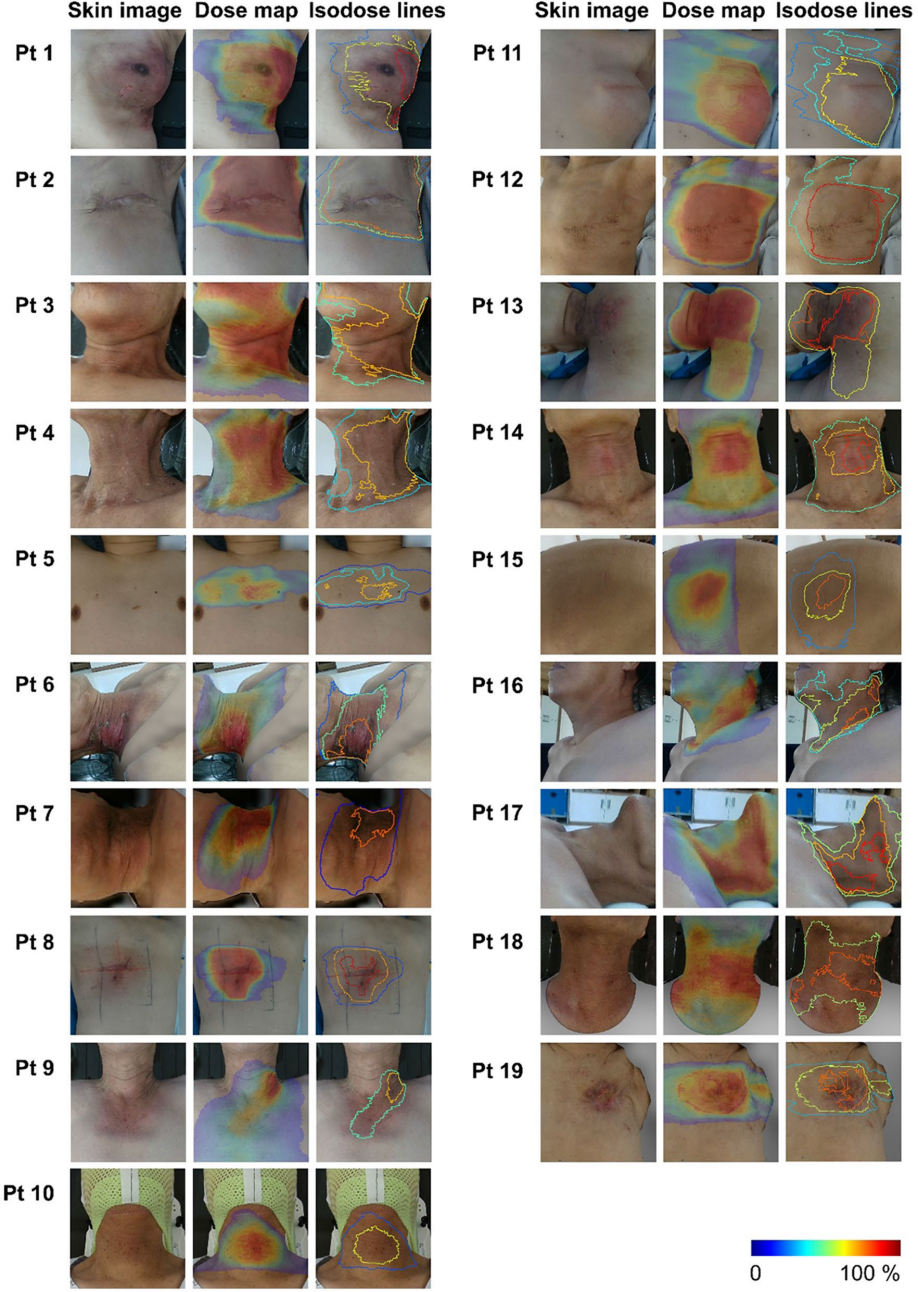
Visualized dose distribution on the skin surface image of radiation dermatitis in 19 patients who underwent radiotherapy. Dose distribution map and isodose distribution are overlapped on patient’s skin image as accumulated dose values up to the time of the 3D scan. The visualized dose map has relative color scale in each patient within dose range exceeding 5 Gy. The isodose curve corresponds to the nearest dose values from the border of radiation dermatitis in each patient.

All patients exhibited RD by treatment termination. Sixteen of 19 patients had acute RD symptoms that were clearly distinct from normal skin on 3DSSI (Table 2). Representative images of the visualized dose distribution integrated with the RD image in each of the 19 patients are shown in Fig. 1. For all patients, we were able to match 3DSD and 3DSSI using RaTES and to visualize the dose distribution on the patient's RD image. Moreover, the relationship between the planned dose distribution and RD pattern could be intuitively evaluated. In all cases, the visualized radiation dose values on the 3DSSI were applied as cumulative dose values until the 3DSSI was acquired.

**Figure 2:**
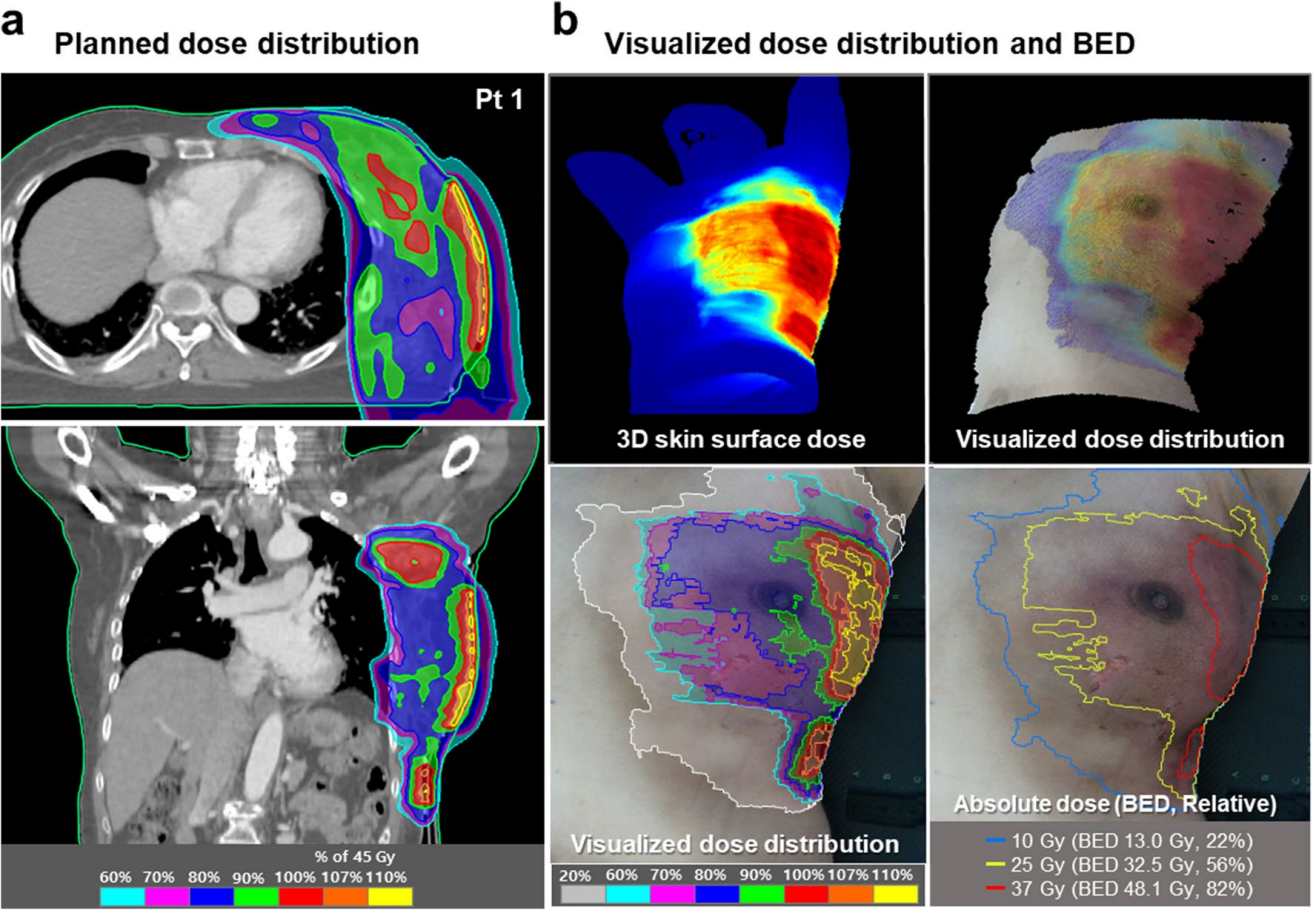
Representative example of a dose image projected onto the patient`s skin (with different dose indices). (**a**) Planned dose distribution in the transversal (top) and coronal (bottom) views from the treatment planning system (TPS) for Patient 1. (**b**) Radiation dose-toxicity evaluation system (RaTES) visualizes the dose distribution at the skin surface in three-dimensional space (top). The physical dose distribution is converted to absolute dose, biologically effective dose (BED), and relative dose. The erythema and moist desquamation are consistent with the pattern of the dose distribution map above the 10 Gy and 37 Gy absolute dose (bottom).

Figure 2 demonstrates one case of an RD patient covered with a dose distribution map that emphasizes three different dose indices: the absolute dose, BED, and relative dose as a percentage of the prescribed dose. The dark skin tones and faint dry desquamations (Grade 1/2) in Fig. 2 are consistent with the shape of dose distribution map above the 10 Gy absolute dose, 13 Gy BED, and 22% relative dose. The moist desquamation in areas other than skin folds (Grade 3) occurs with the dose boundary above the 37 Gy, 48.1 Gy, and 82% for the absolute dose, BED, and relative dose, respectively (Fig. 2). In 16 patients, the dose threshold for RD related to erythema, dry desquamation, and moist desquamation were 5, 18, and 37 Gy, respectively.

The registration accuracy of the ICP algorithm was assessed for all 3DSSIs. The mean deviation between dose PC and 3DSSI PC was 0.27 ± 0.7 mm in the sagittal axis, -0.03 ± 0.7 mm in the horizontal axis, and 0.02 ± 0.3 mm in the longitudinal axis.

## Discussion

This study aimed to develop an intuitive system that can objectively analyze the direct correlation between the radiation dose distribution and RD, by visualizing the absolute skin dose calculated by the TPS on a skin surface image acquired with a 3D camera. To the best of our knowledge, there have been no similar publications on RD in the field of radiation oncology.

Heterogeneous dose distribution and partial hot spots are among the most important predictors of RD^9,16^. However, dose-predicting factors, such as mean doses and the dose-volume parameter^9,16,21^, are not sufficient to explain the RD pattern according to the heterogeneous dose distribution within the same irradiation field. To solve this problem, we developed this new, intuitive radiation dose-toxicity evaluation system, by which the 3DSD can be extracted from the TPS dose distribution and matched with the RD image. Using 82 3DSSIs of 19 patients who underwent radiotherapy, we verified that this new method is very useful in assessing a direct correlation between skin dose distribution and RD pattern.

As 3D cameras provide both photographs and 3D depth data of the body, they are suitable for both visual inspection and matching of RD with the 3D dose distribution. A 2D photograph is hard to match with a 3D dose distribution because there is no information on angle and distance. Hence, we used a 3D-based registration using skin surface data measured by a depth-sensing technique. We used an affordable and portable 3D camera, which was able to create a highly accurate and reliable 3D skin surface model easily. Recently, some studies reported using 3D cameras to measure various changes in the skin caused by radiotherapy^22,23^. These showed that 3D cameras are superior to conventional photographs in detailed assessment of the occurrence and development of skin changes, and in measuring and documenting RD objectively. Currently, 3D camera research is aimed to objectively document RD. In this context, the RaTES matches and visualizes the dose distribution to 3DSSI as a first step toward the development of a comprehensive RD assessment tool. Our novel approach can be used for the accurate prediction and prevention of RD based on TPS dose distribution.

Commercial TPS has limited accuracy in calculating skin surface doses. The accuracy of the skin dose calculation by the TPS depends on the dose calculation algorithm and treatment technique and is therefore different for each institution^17,24,25^. The variability of skin dose calculation suggests that it is necessary to verify the correlation of the skin dose calculated from the TPS of each institution to equally apply the dosimetric indices—or normal tissue complication probabilities (NTCPs)—related to RD severity. The RaTES introduced in this study can be easily applied in institutions, and the contribution of the dose distribution to the onset of RD can be accurately evaluated by matching the RD image with the skin dose distribution calculated by the institutional TPS. This approach can also provide an opportunity to establish institution-optimized skin dose-volume constraints for treatment planning.

The advantage of this approach is a detailed dose distribution map for the degree of RD. Combined 3DSD distribution with 3DSSI showed that a heterogeneous dose distribution leads to severe toxicity at hotspots compared with slight symptoms at low-dose areas (Fig. 1). Further, the dose information visualized on the skin surface images can help to identify non-dosimetric factors that affect the severity of RD. Radiation dose resulting to RD, such as hyperpigmentation, erythema, and moist desquamation, can vary widely among individuals. Some studies showed that concurrent chemotherapy, psoriasis, and smoking history are associated with the occurrence of RD^21,26^. Information obtained can help to identify such clinical risk factors of RD.

Predicting and estimating the radiation dose-toxicity relationship is important for physicians and patients to make informed decisions, as RD affects patients’ quality of life^27^. Currently, dosimetric factors, such as point dose-volume, mean doses, and dose-volume histograms, predict RD. In IMRT/VMAT with a heterogeneous dose distribution, this approach is not effective to demonstrate a direct correlation between dose distribution and RD. If the contribution of the pattern of dose distribution in the TPS to RD can be objectively evaluated, it is possible to predict and manage RD based on the patient's treatment plan and to provide appropriate guidance to the patient. Even if treatment plan adjustment is necessary due to severe RD, the dose distribution can be re-adjusted because the TPS dose and location at which RD is likely to occur can be easily identified. This study provided a basis for establishing a direct correlation between the skin dose distribution and RD image.

Our findings can be applied in various clinical settings. Bernier et al. presented revised comprehensive consensus guidelines for RD in patients with head and neck cancer receiving epidermal growth factor receptor inhibitors in combination with radiotherapy and defined the degree of moist desquamation as the percentage within the irradiation fields^28^. These results allow the clinical application of this grading guidelines, as the RD image and dose distribution can be matched and the irradiation area can be defined as the area receiving a specific dose.

Stereotactic radiosurgery, hypofractionated radiotherapy, and particle therapy have recently been introduced into clinical practice. Establishing a predictive model of RD for a new treatment technique is challenging. Our approach allows an easy and simple evaluation of the correlation between the dose distribution and RD. This can help identify dosimetric factors associated with RD occurrence before establishing a toxicity model for a new treatment technique. In particle treatment, such as proton therapy, the skin can receive a relatively high dose as compared to photon therapy, and the calculation of the skin dose in TPS may be incorrect due to the uncertainty of the relative biological effectiveness (RBE)^9,29,30^. RBE depends not only on linear energy transfer, but also on dose and tissue type. Differences in RBE cause differences between the TPS dose and the dose delivered to the patient. In vivo data are needed to clinically identify RBE differences^31^. The proposed RaTES provides the visualized information of the skin dose distribution matched with patient's RD image, which is useful for verifying the dose and biological effect on the skin related to the occurrence of RD in particle therapy. In addition, the difference in RBE can be indirectly confirmed by cross-comparison with RD- related dosimetric factors of photon therapy.

The present study is the first step toward developing comprehensive and automated assessment tools for RD and has a number of potential applications. In the future, the accuracy and reliability of predictive models may be improved by implementing an automated system that can evaluate RD grading and can generate NTCP suitable for the specific institution and treatment technique. The 3D camera can measure both RD and also various changes to the skin, such as psoriasis, swelling, and fibrosis^22^. Some studies have reported psoriasis as the only clinical factor predictive of RD^21^. We hope to develop an RD prediction system that considers both patient-related factors and dosimetric characteristics using single 3D image acquisition.

There were some limitations to this study. First, the 3DSD and 3DSSI were matched using a rigid image registration algorithm. We used RD images acquired in the treatment position to minimize the error of registration. Further research on deformable image registration is needed to enable accurate matching, even if it is different from the treatment position, as RD increases even after completion of radiotherapy. Second, in the case of two or more plans created during the treatment period for the same treatment site, the doses were deformed and summed to calculate the skin dose accurately. However, changes in dose distribution due to anatomical changes occurring during treatment were not considered. Third, our study focused on a methodology to match and to visualize the skin dose on the patient`s skin image, and the spatial accuracy of 3DSSI and 3DSD was calculated as the mean deviation of registration. However, the agreement between RD pattern and dose distribution was evaluated qualitatively. Since the RD occurrence area is visual information, not quantitative information (segmented data), it could not be analyzed with similarity coefficients or statistical methods. Further study is needed to segment the RD area from the photography. Finally, our findings were based on a limited number of patients from a single institution, which may affect the robustness of the system and may hamper the generalizability of our findings. In particular, further studies are needed with a larger number of patients in a prospective design to investigate the correlation between skin dose distribution and RD using the proposed study approach.

This study visualized the TPS dose distribution on a skin surface image of RD obtained using a 3D camera in patients undergoing radiotherapy. We demonstrated that this new method can intuitively and objectively evaluate the correlation between skin dose distribution and RD directly, reliably, and easily. This approach can also help in the prediction and management of RD caused by heterogeneous dose distribution in IMRT/VMAT. This study forms the basis for the development and validation of a more accurate institution-specific normal tissue complication probability model for RD, and can also support physicians in making treatment decisions based on detailed evidence. However, the results need to be interpreted with caution given the limitations of this study.

## Methods

### Patient and treatment characteristics

We evaluated 19 consecutive patients who underwent radiotherapy at our institute between September 2020 and November 2021. Among the included patients, 15 and 4 were retrospectively and prospectively evaluated. This study was approved by the Institutional Review Board of the Yongin Severance Hospital, and all methods were conducted according to relevant guidelines and regulations. It was based on a review of retrospective charts of patient who received radiotherapy and developed RD. For 15 patients included retrospectively, Informed consent waiver was obtained from Yongin Severance Hospital (9-2020-0163), considering the retrospective nature of the study and the use of anonymized data. We certify that Fig. 1 in the results section was include in the institution-approved waiver of consent. Written informed consent was obtained from the 4 prospectively enrolled patients (9-2020-0120).

Treatment planning was performed using the RayStation TPS (v8.1.2.5, RaySearch Laboratories, Stockholm, Sweden). We used the collapsed cone convolution (version 5.0) and electron Monte Carlo algorithm (ElectronMonteCarlo v3.4) as dose calculation algorithms for photon (volume modulated arc therapy and three-dimensional conformal radiotherapy) and electron beams. The dose calculation grid size was set at 2.0 × 2.0 × 2.0 mm^3^. Treatments were delivered using a linear accelerator (Versa HD, Elekta, Crawley, United Kingdom). All patients were prescribed a moisturizing cream containing physiological lipid granules (Atobarrier cream MD, Aestura, Korea) as a general preventive measure for radiation dermatitis (RD) during the treatment course, according to the institution's standard skin management strategy. Patient characteristics are summarized in Table 1.

### Data acquisition: 3D skin surface imaging and 3D skin dose

The 3DSSI data acquired included the 3D skin surface shape and optical imaging of the area where RD occurred, using an RGB-D camera (Intel RealSense Depth Camera D435i, Intel, Santa Clara, CA), which can simultaneously scan color and depth images. The camera obtained depth data with an accuracy of 2% at 2 m, using active infrared stereo technology based on the time-of-flight principle. The depth and color images were detected with a field of view 86° × 57° and 64° × 41°, respectively, and were synthesized at the same frame^32,33^. The 3DSSI scanned by the depth camera was stored with the color information as a form of point cloud (PC), which is the set of data points comprising height, width, and depth. The skin surface data of the patient were scanned with the patient in the treatment position in the treatment room to minimize the deformation between simulated and scanned positions. The depth camera was rotated around the treatment field during patient scanning to obtain the skin surface data from various angles. The 3DSSI was obtained once per week and on the day of the last treatment fraction. The PC and RGB images were extracted from the recorded scanning files using the Intel RealSense software development kit 2.0. Non-skin surfaces, such as background, treatment table, and immobilization devices, were removed using a semi-automatic algorithm based on the seed point of the skin surface.

**Figure 3:**
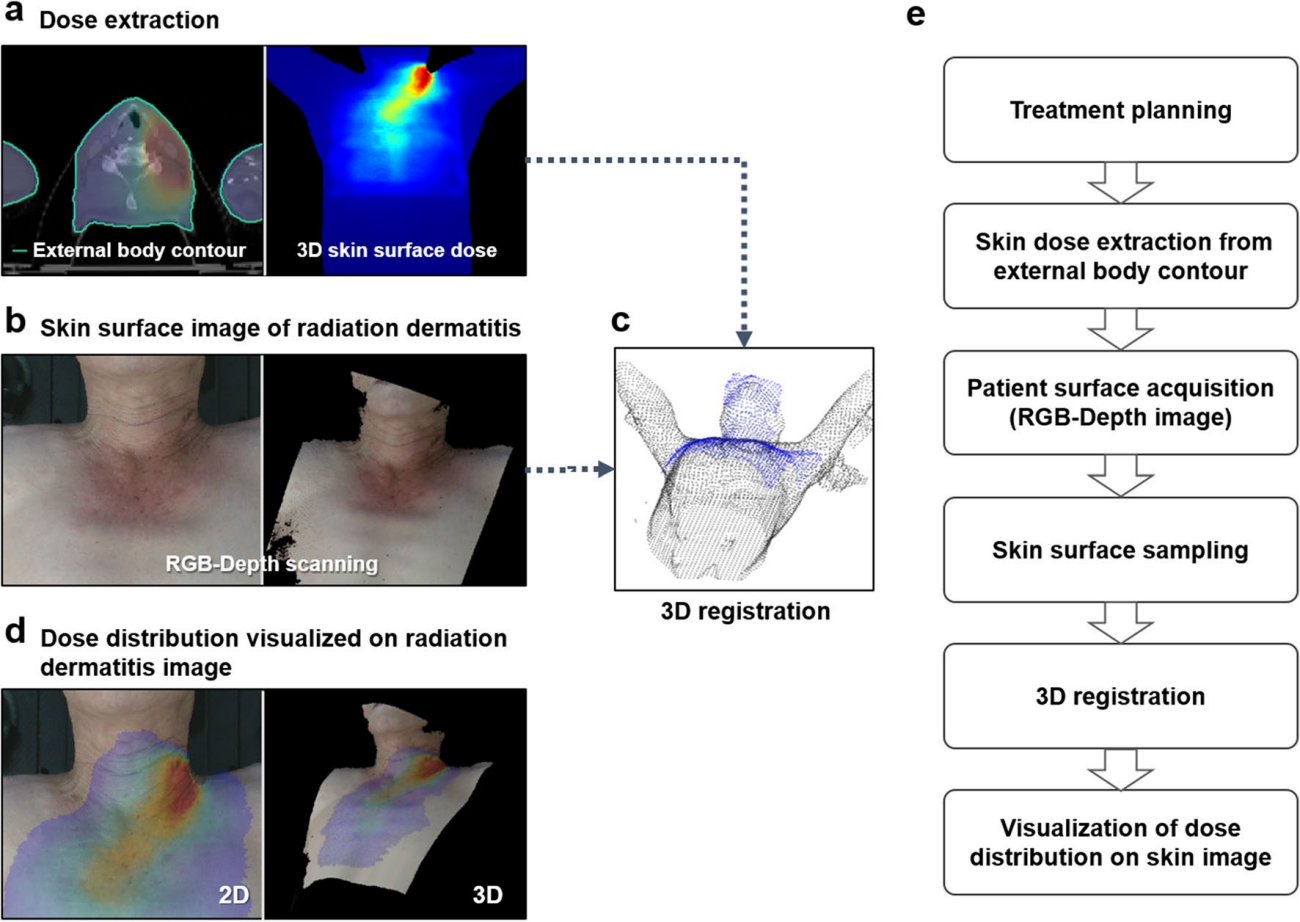
Schematic diagram demonstrating the methodological workflow of the radiation dose-toxicity evaluation system (RaTES). (**a**) Treatment plans are exported in DICOM-RT format for each patient. The 3D skin dose (3DSD) is automatically extracted through the RaTES using RT plan, RT dose, and external body contour, and it is calculated as the cumulative dose of the treatment fractions until 3D skin surface image (3DSSI) is obtained. (**b**) The 3DSSI data of the patients are scanned in the treatment position using an RGB-depth camera, which can simultaneously scan color and depth images. (**c**) Both 3DSD and 3DSSI are registered using an iterative closest point algorithm based on the form of the point cloud dataset. (**d**) The registration outcome is visualized as a 3D surface and a 2D projection image. (**e**) Schematic overview of the workflow conducted in this study.

To project the 3DSD distribution on the 3DSSI, the treatment plans, including the RT-Plan, RT-Dose, and RT-Structure, were exported in DICOM (Digital Imaging and Communications in Medicine)-RT format for each patient. The 3DSD was automatically extracted through the RaTES using RT-plan, RT-dose, and external body contours in RT-Structure. The skin surface of the patient was defined from an automatically generated external body contour using a threshold of -350 Hounsfield Units in computed tomography. The 3DSDs were defined as a set of dose points located at the contour of the skin surface. The dose value of each point of 3DSDs was replaced with the average dose between the point of external body contour and that of 4-mm below the surface to consider skin thickness and dose grid size. The extracted skin dose was converted to a PC dataset comprising dose intensity and position in a 3D Cartesian coordinate at each skin point. Figure 3 summarizes the study workflow and methods.

### RaTES

The RaTES was developed to match the 3D dose distribution calculated by the TPS directly with the 3DSSI of RD patients. The 3DSD was calculated as the cumulative dose of the treatment fractions until the time that the 3DSSI was obtained. Surface registration between 3DSD and 3DSSI was performed using iterative closest point (ICP) algorithm^34,35^. The ICP is an algorithm commonly used for optimizing rigid transformation matrices based on the least squares error. This study applied a point-to-point ICP algorithm embedded in MATLAB version 2020b for the 3DSD to 3DSSI registration. Both PCs of 3DSD and 3DSSI were converted to centimeters, to adjust the scale difference with the dose dataset. Rotation and translation matrices were optimized using the ICP algorithm under the condition of tolerance of error of 0.5 mm and with a maximum of 1000 iterations.

In the RaTES, the data points of the 3DSD PC were removed up to 8 cm above the treatment table, because the skin surface bordering the treatment table could not be detected using the depth camera. Point sampling with a dose cut-off value of 5 Gy was applied to ignore the registration error in the low-dose areas. Moreover, simple truncation was performed in the skin PC to reduce the scanning noise occurring at the surface boundary. Uniform point sampling was performed on both 3DSD and 3DSSI to improve the efficiency of the registration algorithm.

The 3DSD visualized on the 3DSSI were reconstructed as two-dimensional (2D) color images after the 3D skin-dose registration. Each point of the 3DSD was paired with a point on the 3DSSI using the nearest-neighbor search algorithm. To trace the dose information on the 2D skin image, the PC data were converted to a 2D depth image, using a ray-tracing algorithm based on the metadata of the depth camera position. Speeded-Up Robust Features matching was performed between the 2D depth image and optical image to compensate for the difference in viewing angles of the camera sensors. Dose information was overlapped on the optical skin image at the same view location. In addition, RaTES was designed such that the dose intensity of the 2D dose image can be converted to a biologically effective dose (BED) in terms of pixels, according to the equation, BED = n × d [1 + d/(α/β)], where n is the number of fractions, d is the dose per fraction, and α/β is inherent radiation sensitivity value for skin tissue in question. The α/β value for the skin surface was set to 10 Gy^36,37^.

### Data analysis and qualitative evaluation of RaTES

We analyzed 82 3DSSIs obtained from 19 patients who underwent radiotherapy using RaTES. The results of the 3DSD-3DSSI integration of each patient as analyzed by RaTES were qualitatively evaluated by an experienced radiation oncologist and medical physicists. The dose thresholds associated with erythema, dry desquamation, and moist desquamation were evaluated by analyzing the dose distribution visualized in the 3DSSI with the RaTES. In addition, the mean deviation of 3DSD-3DSSI registration was calculated to assess the spatial accuracy of the registration of the 3DSD and 3DSSI.
